# Should breast reconstruction and breast oncoplastic procedures be performed during the coronavirus pandemic?

**DOI:** 10.3332/ecancer.2020.1041

**Published:** 2020-05-11

**Authors:** Raghavan Vidya, Isabel T Rubio, Régis Resende Paulinelli, Alberto Rancati, Agnieszka Kolacinska-Voytkuv, Marzia Salgarello, Hilton Becker

**Affiliations:** 1 Honorary Senior Lecturer, Birmingham University, and Consultant Oncoplastic Breast Surgeon, The Royal Wolverhampton NHS Trust, Wolverhampton, UK; 2 Breast Surgical Oncology, Clinica Universidad de Navarra, Madrid, Spain; 3 Mastology Program, Federal University of Goias, Araujo Jorge Hospital, Goias Anti-Cancer Association, Goiania, Brazil; 4 Henry Moore Oncologic Institute, Universidad de Buenos Aires, Buenos Aires C1053, Argentina; 5 Medical University of Lodz, Lodz, Poland; 6 Università Cattolica del Sacro Cuore, Department of Plastic Surgery, University Hospital A Gemelli, Rome, Italy; 7 VCU College of Medicine—Inova Branch, National Center for Plastic Surgery, McLean, VA 22102, USA

**Keywords:** COVID-19, breast, cancer, reconstruction

## Abstract

The onset of the COVID-19 pandemic has changed the face of the treatment of breast cancer and breast reconstruction globally. Mastectomy with immediate implant-based breast reconstruction was on the rise due to advances in meshes and implants. However, due to the prioritisation of the critically ill and diversion of the work force, breast cancer treatment has drastically changed. This is an opinion paper written by the authors with experience and importance in the scenario of breast reconstructive surgery. The authors are from different countries with the COVID-19 pandemic in different stages.

## Introduction

Breast cancer is the most commonly occurring cancer in women with over 2 million new cases diagnosed per year [1]. Approximately, about 270,000 new cases of invasive breast cancer are diagnosed in the U.S, about 55,000 in the UK and about 560,000 in Europe [2–4]. Surgery remains the mainstay of treatment for breast cancer globally. In England, about 40% of those diagnosed with breast cancer undergo mastectomy as their primary therapeutic procedure [5]. Immediate breast reconstruction is on the rise with implant reconstruction being the most common method due to advances in meshes and implants [6, 7]. Immediate breast reconstruction has the advantages of improved body image, health-related quality of life and patient satisfaction. There is evidence to suggest that women who have immediate reconstruction are more satisfied with the outcome compared to those who opt for delayed reconstruction [8]. This could be due to not having to go through a second operation, which results in further interruptions to their lifestyle. In certain cases, a delayed reconstruction may be mandated to accommodate radiation treatment, which can add to patients’ anxiety. Patients, who have a delayed reconstruction, are more likely to report feelings of anxiety and depression and their body image and self-esteem is inferior to those who had an immediate reconstruction.

## Changes in current practice

The recent onset of the global COVID-19 pandemic [9] has changed the practice of breast oncological surgery. It has major impact on the diagnosis and the treatment of breast cancer due to the focus on critically ill patients. Hospitals in the UK, Europe and USA have restricted their operating room time to emergency surgeries only. Hospitals are facing a lack of ventilators, reducing the operating theatre resources dramatically. Hospital beds and wards are restricted to COVID-19 patients and only a few beds are being kept for emergency surgery and urgent hospitalisation.

Cancer patients are being assessed on a case by case basis, on multi-disciplinary team (MDT) held by telehealth. Prioritisation of cancer subtypes as stated by most national bodies is sensible for guidance although not definitive. Breast cancer patients are offered various options based on the type of tumour. Options include delayed surgery for low grade tumours and ductal cancer in situ (DCIS), with the assessment of hormone receptor status. Breast cancer surgery should be restricted to patients who are likely to experience compromised survival if it is not performed within the next 3 months. This includes patients completing neoadjuvant treatment, those with clinical stage T2 or N1 oestrogen receptor (ER) positive / progestrone receptor (PR) positive / human epidermal growth factor 2 (HER2)-negative tumours, patients with triple negative or HER2-positive tumours, discordant biopsies that are likely to be malignant, and removal of a recurrent lesion [10]. All risk reducing surgeries for gene mutation and prophylactic mastectomy are not recommended. Patients can start with endocrine therapy until surgery is available with reassurance of evolution by telemedicine. In this situation, the availability of healthcare resources and anticipated availability of resources in the post-operative period are causing reconstruction and symmetry procedures not to be considered.

The COVID-19 pandemic raises capacity issues. Where capacity remains, then surgery should continue. The challenge of any pandemic is to maintain the capacity. All management is multimodal and dependent upon clinical and medical oncology availability—one cannot consider reconstruction/ ‘oncoplastic’ surgery in isolation from this.

In case-by-case evaluations, a shared decision process with the patient needs to take into account the risks of reconstruction, including:

- Longer time in the operating room.
- Need for hospital admission.
- Complications resulting from reconstruction may necessitate further surgery and hospital admission, increasing the risk of viral exposure.
- Delayed or immediate delayed surgery would offer a safer approach.

Procedures requiring prolonged hospital admission should be avoided (free flap autologous reconstruction) but other day case and 24 hours stay procedures when feasible can be carried out. This would ensure reduced caseload burden in the aftermath of the first wave of the pandemic. Some countries (parts of Italy and South America) are advocating and adopting the use of prepectoral immediate implant/expander reconstruction at the first instance. The use of simple oncoplastic procedures including mammoplasty and incorporation of perforator flaps for volume replacement can be performed. In fact ‘oncoplastic’ techniques to avoid the need for mastectomy should be encouraged to enhance less invasive surgery. However, all the authors consider it is important for the patient to be fully informed about the risks and benefits of the procedure taking into consideration the risk of hospital acquired COVID-19 infection.

## Options available in preparation for delayed reconstruction or oncoplastic procedures

In those patients where mastectomy can be delayed, this is the best option. If the patients are having a mastectomy, then scar placement should be considered and planned in preparation for delayed oncoplastic procedures. Drains should be placed not to obstruct further reconstruction. Adding regional blocks should be considered so that patients can be discharged sooner. The perforators should be preserved when planning for possible delayed partial breast reconstruction. The options of lipomodelling can be considered at a later date when safe. In preparing the skin flaps for delayed reconstruction, the surgeon should leave healthy flaps to facilitate later reconstruction. Skin sparing and nipple sparing mastectomies should be performed and closed in the standard fashion. Excision of excess skin should be limited except where viability is of concern. The reconstruction can be undertaken at a later date. It has been shown that delayed reconstruction has a lower complication rate than immediate reconstruction.

Prepectoral reconstruction in the delayed-immediate autologous reconstruction patient leads to significantly lower complication rates and shorter interval between staged surgeries. For palliative surgery for local complications that require a mastectomy, if there is a need to cover the defect, rotational flaps are the preferred method.

## World discrepancies and controversies in official recommendations

The gravity and development of the pandemic is unequally distributed around the world. Some countries, like China, are overcoming the crisis, some western countries are just at the top of the incidence, and others do not have cases yet, or just have a few. Recommendations should be balanced according to specific situations. Obviously, COVID-19 guidelines for triage of breast cancer patients depends on numbers of COVID-19 patients in the country, hospital resources, ICU vent capacity and escalation phase of the COVID-19 trajectory.

The official recommendations from different scientific societies may differ. Just to name a few, the American Society of Breast Surgeons (03-25th-2020) divided breast cancer surgery in priority groups. They stated immediate breast reconstruction as intermediate priority. They suggest preferably the use of tissue expander or implant and defer autologous reconstruction. They also suggest that the recommendations should be taken in the context of each institution’s resources and the prevalence of the COVID-19 pandemic in their region.

During the initial phase of the pandemic, The Association of Breast Surgeons, UK, has recommended mastectomy with no immediate breast reconstruction and to offer delayed reconstruction at a later date. This has changed recently and immediate breast reconstruction using implants can be offered in selective patients but is not routinely recommended.

The Brazilian Society of Mastology launched a statement (03-25th-2020) that still considers immediate breast reconstruction as a fundamental part of breast cancer treatment. They suggest that immediate breast reconstructions, especially those with expander, or implants may be suitable, according to the risk for the patient, and according to the surgeon’s and hospital’s conditions. They also suggest that more complex procedures, like myocutaneous flaps, or delayed reconstructions, refinements and symmetrisation should be postponed. Similar recommendations are advocated by the Polish Society of Surgical Oncology and the Italian Society of Plastic Surgery.

In a more restrictive tone, the Argentinian Society of Mastology suggests postponing all immediate breast reconstructions, except in cases of locally advanced breast cancer, for chest wall closure. They suggest that, exceptionally, in some specialised institutions. Immediate reconstructions with expanders or implants could be allowed.

## Conclusion

Our current recommendation is to keep breast cancer surgical treatment simple during the COVID-19 pandemic. We do understand that the pandemic is at different stages globally and guidelines from the national societies and local availability should be considered in treatment planning of breast cancer patients. Options of reconstruction, autologous and complex oncoplastic procedures should be deferred and considered safe when performed as delayed procedures.

### Key points for treatment planning

- The collaboration of the breast team including oncologists essential for treatment planning
- Patient co-morbidities need to be considered
- Availability of theatre space and personal protective equipment
- Consider simple oncoplastic procedures including incorporation of perforator flaps and mammoplasty
- Immediate implant based breast reconstruction can be associated with higher levels of post-operative infections and a higher readmission rate
- Avoid immediate flap reconstruction and complex oncoplastic procedures
- Avoid prophylactic breast surgery

## Funding declaration

There is no funding to declare.

## Conflicts of interest

Hilton Becker is a consultant for Mentor Corp. All of the other authors have no conflicts of interest to declare.
